# Adjunctive interventions: change methods directed at recipients that support uptake and use of health innovations

**DOI:** 10.1186/s13012-024-01345-z

**Published:** 2024-02-08

**Authors:** Justin D. Smith, Dennis H. Li, James L. Merle, Brennan Keiser, Brian Mustanski, Nanette D. Benbow

**Affiliations:** 1 Department of Population Health Sciences, Spencer Fox Eccles School of Medicine, University of Utah, Salt Lake City, UT USA; 2 Department of Psychiatry and Behavioral Sciences and Institute for Sexual and Gender Minority Health and Wellbeing, Northwestern University Feinberg School of Medicine, Chicago, IL USA; 3 Department of Family Medicine, University of Washington School of Medicine, Seattle, WA USA; 4 Department of Medical Social Sciences, Third Coast Center for AIDS Research, Institute for Sexual and Gender Minority Health and Wellbeing, Northwestern University Feinberg School of Medicine, Chicago, IL USA

## Abstract

**Background:**

Implementation science groups change methods into two categories: (1) clinical, behavioral, or biomedical *intervention* targeting recipient’s health outcomes and (2) *implementation strategies* targeting the delivery system. Differentiating interventions from strategies based on their intended functions is critical to accurately attributing their effects to health or implementation outcomes. However, in coordinating 200+ HIV implementation research projects and conducting systematic reviews, we identified change methods that had characteristics of both interventions and strategies that were inconsistently categorized. To alleviate confusion and improve change method specification, we propose that implementation science should adopt an extant but rarely used term—*adjunctive interventions*—to classify change methods that are distinct from the common intervention/strategy taxonomy.

**Main text:**

Adjunctive interventions as change methods that target recipients (e.g., patients, participants) of a health intervention but are designed to increase recipients’ motivation, self-efficacy, or capacity for initiating, adhering to, complying with, or engaging with the health intervention over time. In two of our published reviews on implementation of HIV interventions, 25 out of 45 coded change methods fell into this gray area between strategy and intervention. We also noted instances in which the same change method was labelled as the intervention (“the thing”), as an adjunctive intervention, or an implementation strategy in different studies—further muddying the waters. Adjunctive interventions are distinguished from other change methods by their intended *targets*, desired *outcomes*, and *theory of action and causal processes*. Whereas health interventions target recipients and have a direct, causal effect on the health outcome, adjunctive interventions enhance recipients’ attitudes and behaviors to engage with the intervention and have an indirect causal link to the health outcome via increasing the probability of recipients’ utilization and adherence to the intervention. Adjunctive interventions are incapable of directly producing the health outcome and will themselves require implementation strategies to effectively impact sustained uptake, utilization, and adherence. Case examples, logic modeling, and considerations (e.g., relationship to consumer engagement strategies) for adjunctive intervention research are provided.

**Conclusion:**

Conceptualizing adjunctive interventions as a separate type of change method will advance implementation research by improving tests of effectiveness, and the specification of mechanisms and outcomes.

**Supplementary Information:**

The online version contains supplementary material available at 10.1186/s13012-024-01345-z.

Contributions to the literature

- We formalize the definition of adjunctive interventions, a concept that has appeared in the literature without full explication as to their function, goals, and outcomes, particularly in the context of the broader implementation science literature.
- We provide guidance on distinguishing between three change methods in implementation science: health interventions, implementation strategies, and adjunctive interventions.
- This article also presents a rationale for why understanding the concept of adjunctive interventions, as distinct from health interventions and implementation strategies, would improve research into understanding the theory and mechanisms of action in complex implementation studies and practice-based initiatives.

## Introduction

Implementation scientists have typically grouped change methods^1^ [1] into two categories: (1) an *intervention* being implemented, defined as a clinical, behavioral, or biomedical innovation designed to improve recipient’s (client/patient) health outcomes (e.g., prevent disease occurrence, reduce severity of disease, improve quality of life) [2], and (2) *implementation strategies*, defined as actions taken to improve utilization of an intervention at the health system or provider level (e.g., policy change to improve reimbursement, provider training, provider audit and feedback, and colocation of services) [3]. Clearly differentiating the health intervention (clinical, behavioral, biomedical) from implementation strategies based on their intended functions is critical to accurately attributing the effects of each component on its respective health or implementation outcome [4] and to ultimately understand success or failure in achieving desired results [5]; yet, the distinction remains a source of debate and confusion in the field [6]. Accordingly, thought leaders in the field have developed guidance for researchers to differentiate these change methods, such as Curran’s [7] pragmatic and widely adopted heuristic of “the thing” that is being implemented (i.e., the health intervention) and “the stuff we do to try to help [others] do the thing” (i.e., implementation strategies). Implementation scientists also implement other objects with scientific evidence that might not strictly be a health intervention as we have defined it (e.g., policies, programmes, devices, innovations) [8]. Our perspective may apply to these other "objects" of implementation, but is most germane to those that align with our definition.

In published and ongoing HIV implementation research studies, however, our research team has consistently encountered varying classification and labeling of interventions and strategies. First, while conducting several systematic reviews of implementation-related studies [9, 10], we noticed the same change methods being referred to as a strategy in some studies and an intervention in others; upon closer examination, these change methods did not fit squarely into either of the aforementioned definitions. Peer navigation, for example, is a commonly used change method to increase uptake of pre-exposure prophylaxis (PrEP) among men who have sex with men and has been deemed both a strategy [11] and an intervention [12]. Despite targeting recipients and not the health delivery system, like an intervention, the intended outcome is more closely related to delivery (i.e., uptake) than having a direct clinical outcome. Another example is digital health interventions (e.g., text messaging, apps), change methods designed to support continued use of health interventions (e.g., PrEP (https://www.thecommunityguide.org/pages/tffrs-hiv-prevention-digital-health-interventions-improve-adherence-hiv-pre-exposure-prophylaxis.html), HIV treatment [13]) among recipients, which were frequently studied as “the thing” to be implemented and tested. In our two currently published reviews, one examining PrEP implementation [14] and the other HIV treatment [10], 25 out of 45 coded change methods fell into this gray area between strategy and a health intervention.

Second, in our work supporting implementation research in more than 200 HIV implementation research projects [15] using the Implementation Research Logic Model (IRLM) [16], which visually differentiates interventions and strategies, we have continuously observed HIV researchers struggle to categorize the change methods they are studying. Patient education programs have been a popular focus of these studies and have understandably taken the role of the “the thing” in IRLMs. When mapping the theory of change from patient education to an HIV clinical outcome, however, other biomedical interventions (e.g., PrEP, HIV treatment) inevitably mediate the causal logic, leading researchers to question whether their “thing” is really a strategy. Some of this confusion appears to flow from the discovery of highly effective biomedical approaches to HIV prevention (i.e., PrEP) and the resulting shift of behavioral scientists’ efforts from creating interventions that reduced HIV transmission by changing risk behaviors to applying those same behavioral tools to support uptake and adherence to PrEP.

In this debate paper, we propose that implementation science should adopt an extant but rarely used term that can clarify the precise function and intended effects of these “gray” change methods: *adjunctive interventions*. This term has been found in the literature, including in implementation research studies [17, 18], related to outcomes of both behavioral and biomedical/pharmacologic interventions [19–22], but it lacks clarity and consistency in its use. Thus, we aim to (a) define adjunctive interventions in relation to implementation strategies and health interventions, (b) make the case for why this distinction is needed due to the pitfalls of maintaining the dichotomous intervention–strategy status quo, (c) describe how to specify them, and (d) discuss implications of adding this term to the implementation science lexicon. Conceptualizing adjunctive interventions as a distinct type of change method and ensuring its consistent use will advance implementation research by improving specification of mechanisms and attribution of implementation and participant health outcomes, which will accelerate the production of generalizable knowledge to achieve public health impact.

## What are adjunctive interventions? Definition and key characteristics

**Fig. 1:**
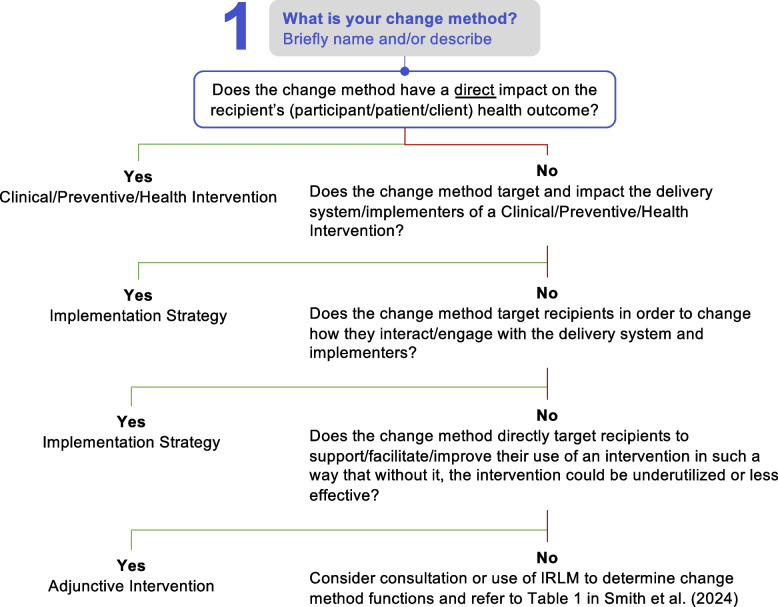
Decision tree for identifying health interventions, implementation strategies, and adjunctive interventions

We define adjunctive interventions as change methods that target recipients (e.g., patients, clients, program participants) of a health intervention and are designed to increase recipients’ motivation, self-efficacy, or capacity for initiating, adhering to, complying with, or engaging with the health intervention initially (uptake) and over time (adherence). To be adjunctive is to be supplemental, in that they support getting to health outcomes but are not always necessary and are never sufficient. They are distinguished from other change methods by their intended *targets*, desired *outcomes*, and *theory of action and causal processes* (Table 1). Whereas health interventions target recipients and have a direct, causal effect on the primary recipient health outcome, adjunctive interventions enhance recipients’ attitudes and behaviors to engage with the intervention but are not capable of directly producing the primary recipient health outcome (i.e., that requires the intervention); rather, there is an indirect causal link to the health outcome via increasing the probability of intervention utilization. Implementation strategies, in contrast to both, primarily target implementing agents (e.g., clinicians, program delivery staff, leaders) and/or delivery processes and structures, with direct causal impacts on implementation outcomes at the system level (e.g., adoption, fidelity, reach). Based on these characteristic differences, we provide a decision tree to help differentiate them (Fig. 1).

**Table 1:** Key characteristics that distinguish health interventions, adjunctive interventions, and implementation strategies

**Characteristics**	**Clinical/preventive/health intervention**	**Adjunctive intervention**	**Implementation strategy**
**Target**	• Directly influences recipients’ disease/condition outcomes of interest	• Influences recipient behaviors and may be needed to enable interaction with the intervention	• Influences implementer behaviors or the implementing system operations
**Primary outcomes**	• Symptoms, biomarkers, infection, diagnosis, other health outcomes	• Recipient acceptability, uptake, utilization, adherence, completion, maintenance of the intervention	• Implementer adoption, reach, feasibility, fidelity, sustainment of the intervention
**Actions and causal processes**	• Changes targets of disease/condition among recipients • Direct, proximal cause of change to the endpoint health outcome	• Changes recipients’ behavior/motivation/access to and/or enhances/facilitates engagement with the intervention • Distally impacts endpoint health outcome through receipt, adherence, and/or maintenance of the intervention, inert on its own	• Changes the way services and interventions are carried out as a result of implementer actions or system changes • Inert on endpoint health outcome, requires the health intervention to have impact
**Hierarchy/level**	• Delivered by implementers in a delivery system • Requires the use of implementation strategies • Effects for some recipients may be enhanced by adjunctive interventions	• Delivered by a variety of different individuals (e.g., implementers, peers) and is commonly delivered using digital methods (e.g., text message, apps) • Requires the use of implementation strategies to support delivery • May be indicated for only a subset of health intervention recipients	• Actors of implementation strategies are wide ranging and can include individuals in the delivery system, external entities, and others • Supports the implementation of either/both the health intervention and the adjunctive intervention
**Indications**	• Biomarkers/health indicators/risk assessment	• Innovation determinants (client-level barriers/facilitators)	• Implementation determinants (system-level barriers/facilitators)

## Recent case in point

Efforts to mitigate the COVID-19 pandemic with vaccines further highlight the challenges discussed in the “Introduction”. In typical implementation science framing, the vaccine for the SARS-CoV-2 coronavirus that causes COVID-19 illness is the preventive intervention, and the need to scale up vaccination requires implementation strategies. In the USA, the implementation strategies were multifaceted and focused on facilitating recipient’s access via offering vaccinations at typical healthcare locations staffed by nurses (e.g., primary and urgent care), pharmacies, public health departments, community-based organizations, and other entities (e.g., churches) [23, 24]. Financial strategies were also invoked by federal and local governments to eliminate cost-related barriers to recipients, and dissemination strategies were deployed to educate clinicians and the public about vaccine safety and effectiveness [25, 26]. However, as of May 10, 2023, only 81% of the population had received at least one dose, and 70% were fully vaccinated (one or two doses) [27]. The below-target vaccination rates were not due to lack of availability for the vast majority of Americans, as evidenced by only 68% of distributed doses being administered [28]; rather, individual uptake of vaccines was insufficient due to stigma, hesitancy, and misinformation, resulting in the majority of unused vaccines expiring (estimated 1.1 billion doses globally).

Vaccine hesitancy has been a well-documented challenge [29], particularly among African American and Latino populations [30, 31]. Indeed, high-level federal officials—from former NIH Director Francis Collins to senior FDA advisors—conceded that they underestimated the role of vaccine hesitancy and should have done more to support uptake [32, 33]. While dissemination of evidence regarding vaccines, ideally from trustworthy sources, is one approach that could have helped combat poor vaccine literacy, misinformation, and disinformation [29], decades of research have shown that information is necessary but insufficient to change most health behaviors [34]. Instead, many health interventions, including vaccines, require theory- and evidence-based change methods that motivate and capacitate eligible people to use them.

Historically, these recipient-focused motivational and capacity-building change methods were considered a type of health intervention, but no amount of vaccine-motivating intervention alone would afford a recipient protection against SARS-CoV-2 absent actual vaccination uptake. One might consider whether these change methods are implementation strategies, but the fact is that these change methods are intended to produce effects at the individual level, not any of the established implementation outcomes. Moreover, additional strategies would be needed to implement these other change methods. Because misspecification of outcomes may lead to erroneous conclusions, the poor fit of these recipient-focused adjunctive interventions to the current intervention–strategy dichotomy is scientifically problematic, and their unique contribution to maximizing the effectiveness of the intervention to achieve public health benefit warrants differential categorization.

## Examples of adjunctive interventions

Table 2 provides several examples of adjunctive interventions, the interventions they support, and the implementation strategies used to implement them both. We selected adjunctive intervention examples from the literature that aligned with our proposed criteria, complemented with synthetic examples of implementation strategies in instances where the study did not specify them. We provide examples for HIV prevention and treatment, hypertension control, and weight management interventions that were tested in conjunction with a range of adjunctive intervention types, including peer navigation, motivational interviewing, cognitive behavioral therapy, parent training, incentives/contingency management, housing programs, and text messaging/eHealth.

**Table 2:** Examples of adjunctive interventions

**Health intervention**	**Problem addressed by adjunctive intervention**	**Adjunctive intervention**	**Implementation strategies for the health intervention**^a^	**Implementation strategies for the adjunctive intervention**
Antiretroviral treatment (ART)	Poor adherence to ART or missed clinic appointments among viremic clinic patients	Connect4Care [35]: Motivational, informational, and supportive text messages three times per week	Task-shifting from health care workers to adherence support workers to address inadequate human resources	Adapt existing EHRs toflag clients with poor retention and pending care appointments. Develop automated feed from EHR data to text messaging platform
Testing, condoms and PrEP	Low motivation and self-efficacy for testing, condoms, and PrEP among young men who have sex with men (MSM)	Keep It Up! (KIU!) [36]: Online, interactive individual-level intervention offered in community-based settings to increase HIV knowledge, motivate and teach safer behaviors, and instill self-efficacy for PrEP, condom use, and other HIV prevention interventions	Use media to spread the word about HIV testing and prevention services; change testing site to increase access to testing	Skills-based training of staff on client recruitment and retention; audit and feedback to monitor and inform midcourse improvements
HIV testing and care linkage	Latino MSM readiness, stigma, medical mistrust, lack of knowledge of available services	Peer navigation and referral to ancillary services [12]	Develop formal referral and agreement processes between community-based HIV testing organizations and clinical providers	Formal patient navigation program structure, tailored training for patient navigators, integration of patient navigation program into broader clinical/program teams
Medication for opioid use disorder (OUD)	Adherence to methadone, buprenorphine, and/or naltrexone treatment for OUD	Contingency management [37] — Incentives for recipients to promote achievement of OUD treatment-related goals	Revise standard operating procedures to expedite access to treatment; brief provider training that includes case examples and research data	Didactic training, performance feedback, and external facilitation
HIV care	People living with HIV who are homeless or unstably housed face particular barriers to achieving and maintaining optimal care	Supportive permanent housing [38]: Provision of affordable, long-term housing with comprehensive supportive services to enhance management of HIV	Client-centered care model, change clinic workflow to facilitate wrap-around services in one visit, change clinical sites of care in community to increase access	Obtain formal commitments between providers of supportive services and network of apartment buildings, create new clinical care and support team to monitor and respond to client’s needs
PrEP	PrEP medication adherence support	Integrated Next Step Counseling [39]: Participant-centered “check-in” on sexual health protection using motivational interviewing strategies	Alter patient/consumer fees, create new clinical teams that include HIV testing and case management services, develop collaborative practice agreements for pharmacists to prescribe PrEP	Develop training materials and implementation manual, protocol development, dynamic training, continuous quality improvement, train-the-trainer model
Antihypertensive medication	Medication adherence in hypertensive patients	Collaborative care, medication review, and tailored adherence counseling including motivational interviewing and telephone follow-ups, by a pharmacist [40]	Training in collaborative care between prescribing clinician and pharmacist	Pharmacist training in motivational interviewing, workflow change to administer, score, and review questionnaires about medication adherence, fidelity monitoring
Antiseizure medications (ASMs)	Medication adherence for children newly diagnosed with epilepsy	Problem-solving counseling intervention to address the family’s individualized adherence barriers delivered by psychologists/trainees [41]	Electronically monitored ASM adherence that is relayed to clinicians	Training by a licensed psychologist who provided monthly supervision (included review of audio sessions and feedback to interventionists, review of treatment manuals, live role plays for clinical content in sessions, and shadowing experiences with more experienced interventionists)
Cardiovascular disease (CVD) risk assessment and treatment	Help people with rheumatoid arthritis (RA) and CVD risk obtain evidence-based CVD risk assessment and treatment	Peer coaching [42]—an intervention in which a person with RA coaches another person with RA	Training in CVD assessment for patients with RA	Educational materials, peer coach training, group supervision, mock sessions, fidelity rating, and certification of competence

Legend: *ART* Antiretroviral treatment, *ASM* Antiseizure medications, *CVD* Cardiovascular disease, *EHR* Electronic health record, *HIV* Human immunodeficiency virus, *MSM* Men who have sex with men, *OUD* Opioid use disorder (MOUD), *PrEP* Pre-exposure prophylaxis, *RA* Rheumatoid arthritis

^a^Most implementation strategies described in this column was not in the original papers; they are either synthetic examples for illustrative purposes or are referenced in another paper

## When and how should adjunctive interventions be used?

While adjunctive interventions could be applied universally (all eligible recipients receive it regardless of individual risk or behavioral indicators), they might more commonly be selective (offered to a subset of recipients with certain risk factors or specific needs) or indicated (offered only to recipients struggling with adherence, retention, and/or engagement with the intervention) based on factors such as complexity and resource availability [43]. A universal adjunctive intervention for medication adherence could be text message reminders that are automatically sent to anyone on a particular regimen. Conversely, a selective adjunctive intervention would be systematically applied only to individuals who experience a specific health-related social need: For example, provision of housing assistance for people living with HIV has been shown to increase adherence to antiretroviral therapy (ART) medications [44], but not all people with HIV need this type of assistance. Adjunctive interventions are indicated when a recipient is considered at risk of nonadherence to the intervention. In this situation, the adjunctive intervention is part of an adaptive care plan in which adherence is tracked over time and only those recipients with low or nonadherence would receive the adjunctive intervention once this behavior became evident. Selective or indicated rather than universal use of the change method is not necessarily a defining feature of adjunctive interventions, but it is a way in which they might differ from many consumer engagement strategies, which we delve into in a later section.

## Implementation strategies are needed to support deliverly of adjunctive interventions

The examples in Table 2 not only illustrate ways in which the adjunctive intervention supports recipients to remain engaged with the health intervention but also illuminate how implementation strategies for the adjunctive intervention may differ or be similar to those of the intervention. In most circumstances, the intervention and the adjunctive intervention will be adopted and supported together by implementers or an implementing system, and, thus, both require implementation strategies. For instance, peer navigators for PrEP accompany clients to their appointment help navigate the social service and healthcare systems, and send appointment reminders, among other activities. In addition to implementation strategies to deliver PrEP (e.g., tailored training for clinical care teams), the peer navigator program itself would require implementation strategies within the same system (agency, hospital), such as training for peer navigators and integrating peer navigators into clinical care teams. While certain discrete implementation strategies could support both, it is likely that strategies specifically supporting delivery of each will be needed. Scott et al. [17] differentiated the implementation strategies needed to support contingency management as an adjunctive intervention in support of engagement in an opioid treatment program from the strategies needed to support the opioid treatment program itself. Relatedly, there are likely to be implementation outcomes associated specifically with the adjunctive intervention. For example, adoption, reach, fidelity, and sustainment of the adjunctive intervention would be important to measure and report in addition to those of the intervention, as the overall impact of an implementation effort hinges on both the intervention as well as the adjunctive intervention being delivered.

## Logic pathway of adjunctive interventions

**Fig. 2:**
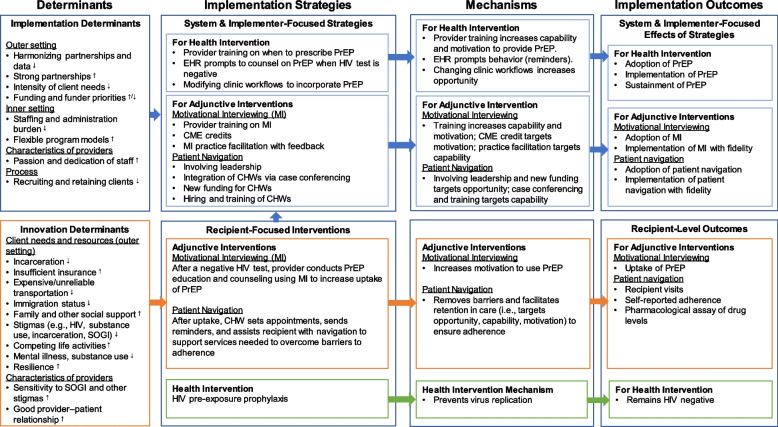
Example implementation research logic model (IRLM) with adjunctive interventions (motivational interviewing and patient navigation) for pre-exposure prophylaxis (PrEP). Legend: CHW, community health worker. CME, continuing medical education. EHR, electronic health record. HIV, human immunodeficiency virus. MI, motivational interviewing. PrEP, pre-exposure prophylaxis. SOGI, sexual orientation and gender identity. ↑/↓Neutral determinant. ↑Determinant facilitator. ↓Determinant barrier

A useful exercise for implementation researchers attempting to differentiate interventions, strategies, and adjunctive intervention is to describe the “logic pathway”. The Standards for Reporting Implementation Studies (StaRI) statement recommends a logic pathway that both describes how the implementation strategy is expected to work to impact implementation outcomes and the mechanism by which the intervention is expected to improve the primary health outcome [45], which is addressed by the IRLM [16]. A modified IRLM can thus be used to clearly depict the different intended effects and hypothesized mechanisms of the intervention, strategy, and adjunctive intervention. Figure 2 provides an example of a completed IRLM for PrEP with two adjunctive interventions: motivational interviewing (MI) [46] and patient navigation. A modifiable IRLM template for adjunctive interventions is included as Supplemental file 1. As previously discussed, implementation strategies are needed to support adoption and delivery of the adjunctive intervention that likely differ but can overlap with the strategies used to support the health intervention. We now discuss each of the remaining elements of the IRLM in relation to the adjunctive intervention.

### Determinants addressed by adjunctive interventions

Adjunctive interventions address one or more of what have been called innovation determinants [47]. Innovation determinants capture recipient-level characteristics and/or experiences with the intervention that predict and/or explain the intended health-related outcomes of the intervention [47]. As such, health-related social needs and/or social determinants can hinder or facilitate the intended health outcomes. For example, a recent review of barriers and facilitators to PrEP identified unemployment, unstable housing, lack of health insurance, complexity of navigating the medical system, and accessibility of PrEP as barriers to PrEP uptake and/or adherence [14]. Similarly, an adjunctive intervention aiming to improve daily adherence to a medication might be based on recipient motivation to change, or addressing health-related social needs, which are an individual’s unmet, adverse social conditions (e.g., food insecurity, housing instability, lack of transportation) that contribute to poor health [48]. Adjunctive interventions that address social conditions could be termed *sociostructural adjunctive interventions*.

### Adjunctive intervention mechanisms

Mechanisms of adjunctive interventions operate through social and/or behavioral factors at the individual (i.e., recipient) level. In the context of a pharmacologic intervention for major depressive disorder, for example, the mechanism of an adjunctive intervention for medication adherence would operate through a recipient-level process (e.g., self-efficacy) that differs from the mechanism of a strategy that aids prescribers in selecting an effective dosage of an antidepressant.

### Outcomes related to adjunctive interventions

The primary outcomes of adjunctive interventions are aligned with the recipients of the intervention. In the case of interventions designed to impact an infectious or chronic disease, the outcome of the adjunctive intervention is behavioral or motivational and impacts recipient’s uptake, adherence, and/or use of the intervention. However, as previously mentioned, adjunctive interventions will often require implementation strategies of their own, and those associated implementation outcomes should be captured separately from the implementation outcomes associated with the intervention. For example, the reach of an adjunctive intervention that only targets participants with poor compliance or attendance would have a smaller denominator than that used for the intervention itself. Adoption and sustainment would be examined separately, as would fidelity of delivery and their respective effectiveness. The other outcomes in the RE-AIM framework [49] would also be applicable to the adjunctive intervention in the same way as they can be applied to the health intervention and to implementation strategies. In essence, it is important to acknowledge that two “things” are being implemented and thus require measurement and reporting of the specific strategies used for each and their respective implementation outcomes. Logic modeling using the modified IRLM (Fig. 2) is a useful method for informing the evaluation plan for studies involving all three change methods.

## Considerations

### The same change method can be an intervention, an adjunctive intervention, or an implementation strategy depending on the target, mechanisms, and outcomes

We previously stated that a defining characteristic of adjunctive interventions is that they do not have a direct impact on the primary health outcome. Yet, it is important to note that the same change method could be an intervention or an adjunctive intervention depending on the target (implementer or recipient), theory of change for the change method, and hypothesized proximal outcome(s). For example, in Table 2, we identify cognitive behavioral therapy (CBT)-based medication adherence counseling as an adjunctive intervention to encourage adherence to ART, yet counseling alone cannot lower viral load unless ART is used consistently. On the other hand, CBT used to treat depression (without medication) can directly change the health outcome (depression symptoms) and thus would be a health intervention rather than an adjunctive intervention. Consistent with Fig. 1, differentiating when the change method is a health intervention and when it is an adjunctive intervention requires examination of the proposed target, function, or theory of change. Logic modeling can also be useful in making this determination.

Occasionally, a change method that is a health or adjunctive intervention could also be an implementation strategy. One example is MI, and Table 3 distinguishes how MI can function in these three change method capacities (intervention, adjunctive intervention, implementation strategy). Although MI as a health intervention is known to influence recipient self-confidence, self-efficacy, and motivation for change in a broad sense, it has frequently been used consistent with our conceptualization of an adjunctive intervention. MI has been used for numerous health conditions in a manner that aligns with our definition and function of adjunctive intervention [50–52], most commonly in the context of medication adherence in chronic conditions (e.g., HIV, hypertension, mental health) [53–55]. For example, MI can be considered an adjunctive intervention when it is being used to target motivation to engage in and adhere to a health intervention, such as antihypertensive medications for blood pressure control [56]. In this example, MI targets recipient uptake of the medication and their continued adherence over time, which controls blood pressure and reduces risk for serious cardiovascular events and heart disease (i.e., the health outcome). MI can also serve as an implementation strategy when it influences implementer behaviors or system operations (rather than recipient’s motivations), and the outcomes are related to implementation. A growing body of evidence indicates that MI with implementers may be effective as a strategy to promote the implementation of evidence-based interventions in health systems [57], schools [58], and other contexts. For example, group-based motivational interviewing has been used to improve general education teacher self-efficacy, adoption, and implementation of an evidence-based classroom management intervention [59].

**Table 3:** Motivational interviewing (MI) as a health intervention, adjunctive intervention, or implementation strategy

**Characteristics**	**MI as the health intervention**	**MI as the adjunctive intervention**	**MI as the implementation strategy**
**Target**	• Directly impacts recipient’s intent to change a health-related behavior	• Can be leveraged to help patients change health-related behaviors, such as HIV medication adherence or reducing substance use	• Can be used as a coaching tool to influence implementer motivations and behaviors to promote evidence-based practices like service integration, new policies, and procedures
**Outcomes**	• Self-confidence and self-efficacy for change	• Positive health behaviors like taking HIV medication daily to achieve viral suppression or reducing substance use	• Adoption, fidelity, reach, or sustainment of an evidence-based practices
**Hierarchy/level**	• Requires implementation strategies (e.g., ongoing training, learning collaboratives, audit and feedback)	• Requires implementation strategies (e.g., ongoing training, learning collaboratives, audit and feedback)	• Supports the implementation of either/both the intervention and the adjunctive intervention
**Indications**	• Low desire or motivation for change, lack of self-confidence, self-efficacy	• Client reports difficulty with medication adherence, substance misuse, etc.	• Relative priority of evidence-based practices among providers is low

Consider the example of treatment for substance use disorders (SUDs). MI has evidence of effectiveness as an intervention that directly reduces likelihood for meeting diagnostic criteria for an SUD [60, 61]. MI also can function as an adjunctive intervention; it has been found to enhance recipient engagement in more intensive substance-abuse treatments [62, 63]. Finally, MI can be conceptualized as an implementation strategy [57], and it has been used as a coaching strategy to impact clinicians’ adoption decisions for alcohol-related intervention, such as screening as well as adherence to clinical practice guidelines and use of educational interventions related to alcohol abuse with patients [64, 65].

## Relationship between adjunctive interventions and consumer engagement strategies

Although implementation research has long examined strategies targeting patients/recipients, these have often been poorly defined and consequently used incorrectly or interchangeably with interventions. Those new to implementation science often assume that strategies target the delivery system, and all recipient-focused change methods are thus interventions. The concept mapping results of the Expert Recommendations for Implementing Change (ERIC) [66, 67] project provided some clarity on this with a category of implementation strategies labeled “engage consumers”. Anecdotally, we (the authors) have observed more confusion about what belongs in this category compared to the other ERIC categories that quite clearly target the delivery system, deliverers, or processes therein. Under our conceptualization of adjunctive interventions, one of the discrete strategies listed within “engage consumers” refers clearly to adjunctive interventions: *intervene with patients/consumers to enhance uptake and adherence* (defined as developing strategies with patients to encourage and problem solve around adherence). The targets are recipients, and the purpose is to support adherence to the intervention. In examining the four remaining strategies in this category, consumer engagement strategies might be understood/redefined to be population focused (i.e., universally applied to all or to a specific group but without distinguishing eligibility or receipt of the intervention), whereas adjunctive interventions specifically target those individuals who have already been reached by the health intervention, are thus assumed to be eligible, and are specifically for uptake and adherence to a health intervention.

**Fig. 3:**
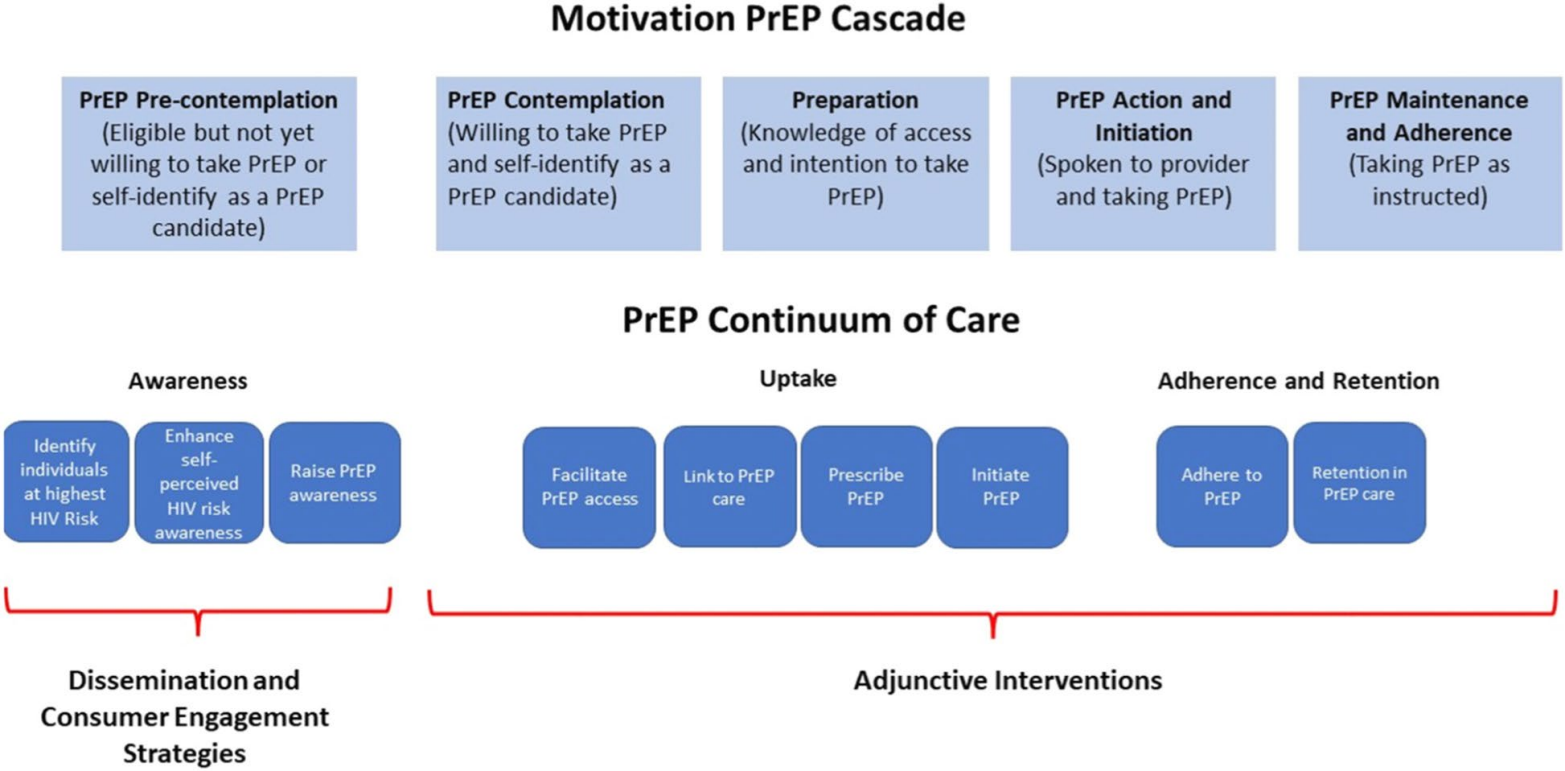
Adjunctive interventions vs. dissemination and consumer engagement strategies along the PrEP motivation cascade and continuum of care. Legend: HIV, human immunodeficiency virus. PrEP, pre-exposure prophylaxis

To further illustrate the different roles consumer engagement strategies and adjunctive interventions can contribute to scaling up an intervention, we examined the motivational PrEP cascade and the PrEP care continuum to show when the use of each comes into play (Fig. 3). To inform people who would benefit from the protection of PrEP, a broad, population-level campaign is most effective to raise awareness, interest, and foster acceptability of PrEP. This is the purview and purpose of consumer engagement and dissemination strategies. If these strategies are successful, individuals are aware of and interested in PrEP but may need help to identify places where they can receive it, financial assistance to pay for it, and/or skills and motivation to adhere to PrEP and stay engaged in care to receive ongoing prescriptions—the exact purview of adjunctive interventions. This example applies to many biomedical interventions, including COVID-19 vaccination as described previously. A taxonomy of adjunctive interventions could be developed to further classify the types that exist, such as text messaging, incentives, and counseling.

## Developing and testing adjunctive interventions

Many change methods that would be defined as adjunctive interventions under our definition have a well-established evidence base, including include several medication adherence interventions, peer navigation, community health workers, contingency management, and some eHealth interventions. However, we have found that many studies testing these adjunctive interventions were not conducted in the broader context of an implementation study where they also examined the implementation strategies needed to support the adjunctive interventions. They also rarely tested the effects of the adjunctive intervention separately from the overall impact on health outcomes that included the effect of the health intervention. Many existing research designs can be used to test the effects of adjunctive interventions. The critical point is to design studies to examine the effects of the adjunctive intervention *separately* from the impact of strategies and the health intervention itself.

## Implications for implementation research and practice

The addition of adjunctive interventions to the implementation science lexicon will benefit researchers and implementation practitioners in different ways. For implementation practitioners, the adoption of the adjunctive intervention terminology could aid organizations in evaluating programs and assessing what is and what is not working well. For example, when program outcomes are lower than expected, one might hypothesize that (a) the intervention does not work well for the population served, (b) implementation of the intervention could be improved, or (c) the intervention works, but participants have poor adherence. If adherence is shown to be low, then an adjunctive intervention could be implemented to address this issue. Without this conceptualization, a decision-maker might elect to either change the intervention or the implementation strategy to achieve the desired outcomes, which could result in greater resource expenditures and not solve the underlying problem.

For researchers, adding adjunctive interventions provides a name and the conceptual underpinnings for better specification of change methods and their functions within a larger study. When seeking grant funding for implementation projects, not being clear about the function of change methods can have negative consequences; it can lead grant reviewers to focus on the wrong aspects of the study in terms of what is innovative (e.g., it may be novel to add an adjunctive intervention to the delivery of an evidence-based intervention or test an implementation strategy for an adjunctive intervention) and significant. We have witnessed instances where implementation science review panels have taken issue with the labeling of a change method, resulting in low impact scores due to that alone. For example, a colleague of the first author (J. D. S.) proposed testing a peer support specialist program as an implementation strategy to improve the patient-level outcomes for a medication-based intervention for schizophrenia. The application was not discussed, as reviewers considered the peer support specialists as an intervention and not an implementation strategy even though the function of the peer support specialists was to support greater uptake and compliance to the intervention. If our proposed conceptualization was used, peer support specialists would be considered an adjunctive intervention, and testing the implementation strategies needed to support its delivery within the medication-based program for schizophrenia could have clarified the innovation, research questions, and significance of the proposal.

Adjunctive interventions as a third category could also provide clarity in funding opportunity announcements (FOAs) and grant applications. In our experience with EHE projects, the FOAs often call for research on the implementation of specific HIV interventions (i.e., testing, PrEP, treatment), and while applicants recognize the importance of adjunctive interventions in successful use of these health interventions, they are often unclear about where these adjunctive interventions fit within the scope of their proposals. Clarifying the role that adjunctive interventions play in supporting the health interventions and the need for implementation strategies to deliver them effectively in FOAs will strengthen both the research proposals and the delivery of the interventions.

Existing theorical models of implementation are useful in understanding the potential contribution of adjunctive interventions in implementation science. Berkel et al.’s [68] theoretical cascade model of implementation depicts recipient engagement, participation, and attendance as the mechanism explaining the relationship between the quantity and quality of intervention delivery and the primary recipient health outcome. Adjunctive interventions would be used to directly target participant engagement, participation, and attendance, while strategies would be tested to support both the adjunctive intervention and the intervention itself. Understanding the impact of the adjunctive intervention and the implementation strategies would thus necessitate outcomes at the recipient and implementer levels, respectively. Mischaracterizing adjunctive interventions as implementation strategies means that researchers might never get to identifying and testing implementation strategies for these change methods. Applying our conceptualization and recommendations concerning adjunctive interventions also has the potential to advance the science in understanding the strategies required to effectively deliver the intervention *and* the adjunctive intervention.

## Conclusions

This debate highlights a critical need for implementation science—clarifying the distinction between different change methods according to their function, targeted individuals, and direct outcomes—by adding the language of adjunctive interventions to the familiar clinical/prevention/health interventions and implementation strategies. We hope the definitions and guidance provided in this paper help implementation researchers, funders, grant reviewers, and implementation partners achieve greater understanding about change methods that fit outside the traditional strategy–intervention dichotomy. We urge investigators and grant reviewers to consider the function and theory of change when determining how to characterize change methods and warn against relying on the ways they have been labeled in the literature thus far. Deploying this new category will necessitate relabeling of previously referenced interventions and strategies, but we believe our conceptualization provides much needed clarity and utility to the field. There will likely be examples of change methods that fail to fall neatly into one of the three categories discussed in this paper, and there will be new considerations and thinking that move the field forward, but we hope this is a valuable heuristic to improve the rigor, reproducibility, and transparency of implementation research.

### Supplementary Information

**Additional file 1.** Implementation Research Logic Model (IRLM) with Adjunctive Intervention.

## Data Availability

Not applicable.

## Abbreviations

ART: Antiretroviral treatment
CFIR: Consolidated Framework for Implementation Research
CHW: Community health worker
EMR: Electronic medical record
ERIC: Expert Recommendations for Implementing Change
IRLM: Implementation Research Logic Model
MI: Motivational interviewing
PrEP: Pre-exposure prophylaxis

## Footnotes

1 “Change methods” were chosen because it was deemed to be the best fitting term to concisely describe health interventions, implementation strategies, and adjunctive interventions. We acknowledge that this term leaves something to be desired as it may fail to fully encompass all types of health interventions. We view health interventions as change methods in that they are aimed at changing the behavior, symptoms, disease status, etc. of the recipient.
