# Supplementary material for: Adjunctive interventions: change methods directed at recipients that support uptake and use of health innovations
Source: Implement Sci. 2024 Feb 8;19:10. doi: 10.1186/s13012-024-01345-z (PMC10854146; doi:10.1186/s13012-024-01345-z)
Supplement: Supplementary file 1 — Additional file 1. Implementation Research Logic Model (IRLM) with Adjunctive Intervention. [file 13012_2024_1345_MOESM1_ESM.pptx]

## Slide 1
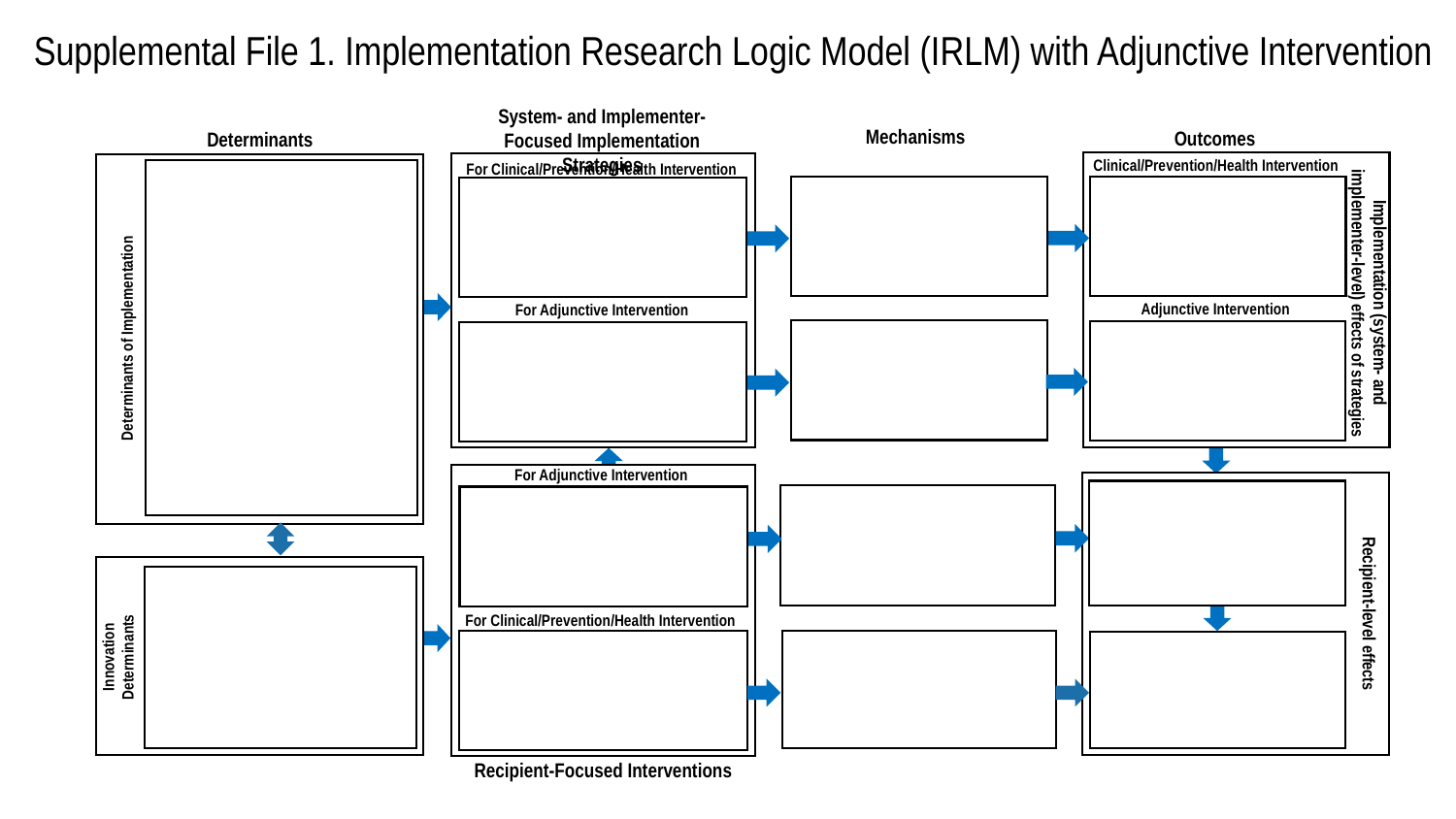

Supplemental File 1. Implementation Research Logic Model (IRLM) with Adjunctive Intervention
System- and Implementer-Focused Implementation Strategies
Mechanisms
Outcomes
Determinants
Clinical/Prevention/Health Intervention
For Clinical/Prevention/Health Intervention
Implementation (system- and implementer-level) effects of strategies
Adjunctive Intervention
For Adjunctive Intervention
Determinants of Implementation
For Adjunctive Intervention
Recipient-level effects
For Clinical/Prevention/Health Intervention
Innovation Determinants
Recipient-Focused Interventions
